# Elevated Serum IL-6 as a Negative Prognostic Biomarker in Glioblastoma: Integrating Bioinformatics and Clinical Validation

**DOI:** 10.7150/jca.104759

**Published:** 2025-01-01

**Authors:** Sup Kim, Kyung Hwan Kim, Hee-won Jung, Eun-Oh Jeong, Han-Joo Lee, Jeanny Kwon, Hyon-jo Kwon, Seung-Won Choi, Hyeon-Song Koh, Seon-Hwan Kim

**Affiliations:** 1 Department of Radiation Oncology, Chungnam National University Hospital, Chungnam National University School of Medicine, Daejeon, South Korea.; 2 Department of Neurosurgery, Chungnam National University Hospital, Chungnam National University School of Medicine, Daejeon, South Korea.

**Keywords:** Interleukin-6, Glioblastoma, Bioinformatics

## Abstract

**Background:** Glioblastoma multiforme (GBM) is the most lethal type of primary brain tumor, necessitating the discovery of reliable serum prognostic biomarkers. This study aimed to investigate the prognostic value of serum Interleukin-6 (IL-6) in GBM patients.

**Methods:** Bioinformatics analysis via gene set enrichment analysis was conducted on The Cancer Genome Atlas RNA-seq data to explore the pathways enriched in samples with high *IL-6* expression. The Tumor IMmune Estimation Resource database was used to analyze the association between *IL-6* expression and immune cell infiltration. To validate the role of IL-6 in a clinical setting, a retrospective cohort study was conducted on newly diagnosed GBM patients. Serum IL-6 levels were repeatedly measured at key milestone time points, and their correlation with survival data was analyzed.

**Results:** Bioinformatics analysis revealed that high *IL-6* expression is associated with the activation of procancer pathways, that there is a positive correlation between *IL-6* expression and immune cell infiltration in GBM. Between March 2021 and September 2023, 36 GBM patients and their serum IL-6 measurements at various time points were included in the clinical data analyses. Elevated serum IL-6 levels at baseline, with a cutoff of 7pg/mL, were identified in 11 patients (30.6%). In the multivariate analyses for overall survival (OS), elevated IL-6 was a significant risk factor (p = 0.048), along with unfavorable surgical resection (p = 0.039) and O6-methylguanine-DNA methyltransferase promotor unmethylation (p = 0.027). The median actuarial OS of the high initial IL-6 group was significantly shorter than that of the low initial IL-6 group (6.4 vs. 19.7 months, p < 0.001). However, IL-6 levels at other time points were not related to patient prognosis.

**Conclusion:** Elevated *IL-6* mRNA expression is correlated with the activation of procancer pathways, increased immune cell infiltration, and poor prognosis in GBM patients. In addition, elevated serum IL-6 at baseline is a negative prognostic factor confirmed in a clinical study. Serum IL-6 may be a potential prognostic biomarker enhancing the management of GBM.

## Background

Glioblastoma multiforme (GBM) ranks as the most lethal form of brain cancer, posing significant challenges in both prognosis and therapeutic approaches 1, 2. Despite advancements in medical science, the prognosis for GBM patients remains predominantly unfavorable, underscoring the urgent need for the development of mechanism-based approaches and the identification of new therapeutic targets and dependable biomarkers to predict the course of the disease 3. Numerous studies have delved into various molecular markers, yet the quest for an unequivocal prognostic indicator is still ongoing 4.

Recently, bioinformatics analysis has been playing a vital role in identifying potential genomic biomarkers more accurately from an enormous number of candidates in oncology department 5, 6. This method can reduce time and cost compared to the wet-lab-based experimental procedures and can contribute to screening tumor-specific genes and prognosis-relevant biomarkers. Currently, microarray and RNA-seq data downloaded from Gene Expression Omnibus and The Cancer Genome Atlas (TCGA) database can be used to correlation between genes transcription expression levels and prognosis 7, 8. Although several studies have described that differentially expressed genes in GBM tumorigenesis via bioinformatic analysis were identified, the prognostic value of these genes has not become widely acceptable in clinical practice. Therefore, identification and application of molecular markers of GBM based on bioinformatic analysis will provide better understanding of therapeutic targets, prognostic judgment and tumor progression.

Interleukin-6 (IL-6) has gained prominence in oncological research because of its critical functions in inflammation, angiogenesis, and the immune response 9. Elevated IL-6 levels have been consistently associated with adverse outcomes across different cancer types 9-11. *In vitro* studies have shown that IL-6 signaling contributes to the development and progression of GBM, including tumor growth, immune system modulation, and metastasis 12, 13. Furthermore, mutant epidermal growth factor receptor-induced IL-6 secretion maintains the active process of tumor heterogeneity in glioblastoma 14. Although these studies shed light on the importance of IL-6, its specific impact on the prognosis of GBM patients requires further investigation.

This study hypothesized that IL-6 could play a pivotal role in predicting the prognosis and clinical course of patients with GBM. First, bioinformatics analyses were conducted using public gene expression data to elucidate the association between *IL-6* expression and GBM prognosis. Next, to achieve clinical validation, this study aimed to determine the prognostic significance of serum IL-6 levels in a prospective GBM cohort.

## Materials and Methods

### Bioinformatics analyses

By employing gene set enrichment analysis (GSEA), we investigated how elevated *IL-6* expression was linked to key oncogenic pathways. GSEA was performed via GenePattern as previously described 15. Briefly, the mRNA-Seq profiles (rnaseqv2 illuminahiseq rnaseqv2 unc edu Level 3 RSEM_genes_normalized) with GBM was obtained from Firehose (https://gdac.broadinstitute.org/). Patients with missing expression values were excluded from further analyses. The patients were then ranked based on IL-6 expression and divided into high (50%) and low (50%) expression groups according to rank, i.e. the threshold was set at the medial IL-6 expression value. Phenotype labels were permuted 1,000 times, and a normalized P < 0.05 and false discovery rate (FDR) of < 0.25 were selected as statistically significant enrichments.

Furthermore, we assessed the correlation between IL-6 expression and immune cell infiltration as well as patient survival via the Tumor IMmune Estimation Resource (TIMER) database. TIMER (http://timer.cistrome.org/) is a comprehensive resource for systematic analysis investigating the associations between immune infiltrates and genetic or clinical features across diverse cancer types 16. We explored the correlation between different tumor immune subsets (regulatory T cells, neutrophils, macrophages, monocytes, dendritic cells and cancer-associated fibroblasts) and *IL6* expression in GBM patients via the TIMER database. Using this database, we also investigated the association between *IL-6* expression and the survival rate of patients with GBM.

### Patient selection, serum IL-6 test and data collection

Since 2021, all patients diagnosed with glioblastoma through tissue confirmation have been prospectively enrolled into the local database and biobank of a single tertiary institution and were assessed for eligibility. Patients who never underwent serum IL-6 testing and were not appropriately followed up were excluded. During the course of treatment, patients are typically subjected to serum IL-6 testing at several time points, including initial (preoperative), pre-CCRT (concurrent chemoradiation therapy), post-CCRT, and thereafter monthly during the administration of adjuvant temozolomide therapy. Blood samples were collected in bottles containing silica particles to facilitate serum separation and were stored at 4°C. The levels of serum IL-6 were measured via an electrochemiluminescence immunoassay (Elecsys IL-6 assay, F. Hoffmann-La Roche Ltd., Basel, Switzerland), with a detection range of 1.5-5000 pg/mL. The reference value for elevated IL-6 provided by the manufacturer is 7.0 pg/mL, which corresponds to the 95th percentile reported in a study conducted on 817 healthy individuals 17. Test results indicating elevated IL-6 levels, potentially influenced by other medical conditions, such as infectious diseases, neoplasms in other organs, liver and kidney dysfunctions, and trauma, were excluded to ensure that increases in IL-6 were relevant to the study's objective. To enhance reliability, serum IL-6 was measured together with routine cell count, liver and kidney function tests, and C-reactive protein (CRP). Patients with CRP > 0.5 accompanied by fever, leukocytosis, or other systemic inflammation were excluded. Additionally, abnormal liver or kidney function test results led to exclusion when clinical symptoms were present. Due to nonspecific rises in IL-6 levels immediately after surgery, multiple measurements were taken to ensure reliable data. In addition, for tests conducted redundantly during the same time period, a lower IL-6 value, which is considered to be measured under stable general conditions, was adopted.

Demographic information, including tumor characteristics, extent of resection (EOR), survival outcomes, and O6-methylguanine-DNA methyltransferase (MGMT) promoter methylation status, was retrieved from a thorough medical chart review. The EOR was classified according to a recent report from the response assessment in neuro-oncology (RANO) resection group and dichotomized into favorable (supramaximal and maximal contrast-enhancing resection) and unfavorable (submaximal contrast-enhancing resection and biopsy) resection in this study 18. The primary endpoint was overall survival (OS), which was derived from medical records and data provided by the national database for academic purposes only. The secondary endpoint was tumor progression following the recently updated criteria from RANO 2.0 19. To examine the possible correlation between IL-6 elevation and tumor progression, the sequential IL-6 values of each patient were plotted on a time-based graph, and the time points of tumor progression were simultaneously marked to check for any correlation. IL-6 elevation was defined in two ways: when the IL-6 value was 7 pg/mL or higher or when the IL-6 value increased more than twice the previous value. The presence of a clinical correlation was defined as follows: tumor progression was observed when there was an increase in IL-6, or pseudoprogression or stable disease was observed when there was no increase in IL-6.

### Statistical analysis

All the statistical analyses were performed via R software (R Project for Statistical Computing), and a two-sided p value of 0.05 was used as the threshold of significance in all the tests. Categorical data are presented as the number of patients with percentages, parametric continuous variables are presented as the means with standard deviations, and nonparametric data are presented as medians with ranges. The Mann-Whitney U test and Fisher's exact test were used to analyze normally distributed categorical and nonparametric continuous variables, respectively. The Kaplan-Meier method was used to analyze progression-free survival (PFS) and OS, with group comparisons performed via the stratified log-rank test. A stratified Cox proportional hazards model was used for univariate and multivariate analyses. Sequential IL-6 data at multiple time points for each patient were presented via multiple line graphs generated with the Matplotlib library of Python (version 3.12, free software).

## Results

### Association of high *IL-6* levels with procancer pathways

**Figure 1:**
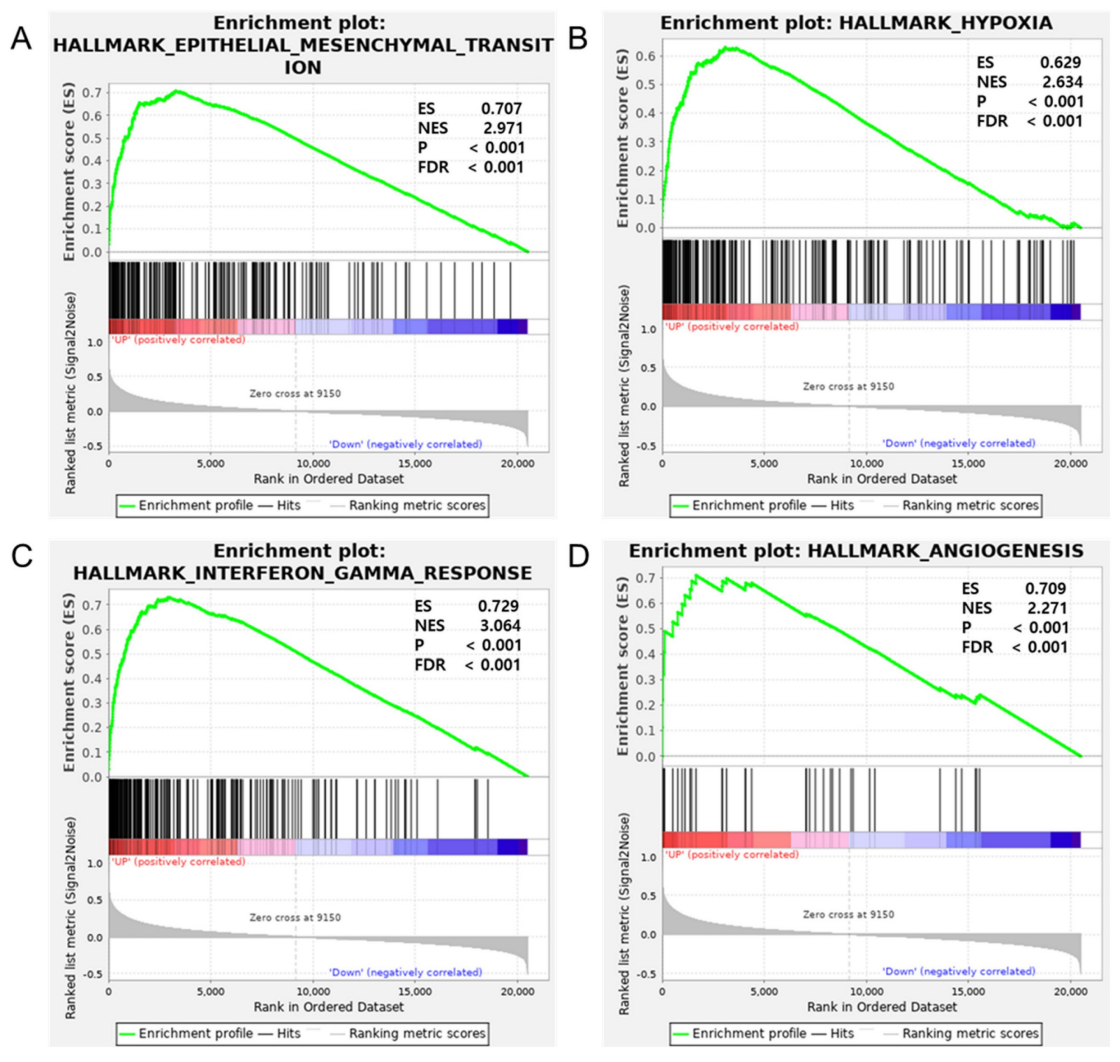
The relationship between *IL-6* mRNA expression and procancer pathways in glioblastoma. Gene set enrichment analysis reveals correlation between *IL-6* expression and genes involved in (A) epithelial-mesenchymal transition, (B) hypoxia, (C) interferon gamma response and (D) angiogenesis. ES = enrichment score; NES = normalized enrichment score; P = p-value; FDR = false discovery rate.

Previous studies have established the role of IL-6 in promoting cancer growth, progression, and immune responses across various cancers 20-23. These studies support the hypothesis that IL-6 may regulate procancer pathways in GBM, influencing the tumor microenvironment (TME). To examine this, GSEA was performed by comparing high and low *IL-6* mRNA expression groups in hallmark gene sets via TCGA GBM mRNA-Seq data (Figure 1). The analysis revealed a significant difference (FDR, < 0.25; nominal P < 0.05) between the two groups with respect to enrichment with genes from the MSigDB Collection (h.all.v6.2.symbols.gmt), particularly in pathways such as HALLMARK_EPITHELIAL_MESENCHYMAL_TRANSITION (normalized enrichment score [NES], 2.971), HALLMARK_HYPOXIA (NES, 2.634), HALLMARK_INTERFERON_GAMMA_RESPONSE (NES, 3.064) and HALLMARK_ANGIOGENESIS (NES, 2.271), which were differentially enriched in the *IL-6* high-expression group. These findings indicate a potential mechanistic link between IL-6 and the exacerbation of oncogenic processes in GBM.

### *IL-6* expression is correlated with immune infiltrates in GBM

**Figure 2:**
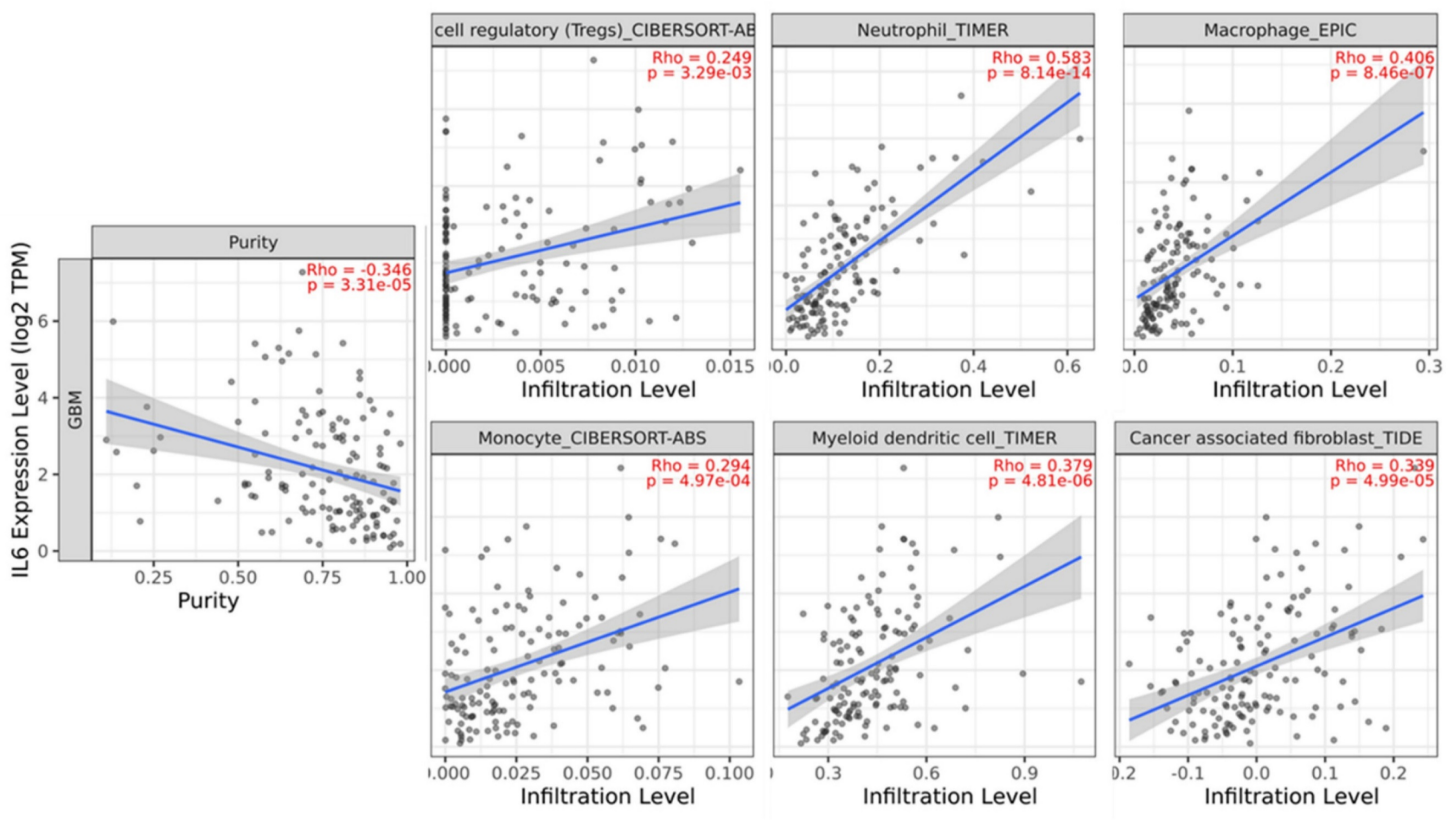
Analysis of the correlation between *IL-6* expression and immune cell infiltration levels in glioblastoma tissue using the Tumor IMmune Estimation Resource (TIMER) database. *IL-6* expression had a significant positive correlation with regulatory T cells (spearman r = 0.249, p = 3.29e-03), neutrophil (spearman r = 0.583, p = 8.14e-14), macrophage (spearman r = 0.406, p = 8.46e-07), monocyte (spearman r = 0.294, p = 4.97e-04), dendritic cell (spearman r = 0.379, p = 4.81e-06), cancer associated fibroblast (spearman r = 0.339, p = 4.99e-05).

Numerous studies have indicated that the procancer pathways identified via GSEA are related to immune cell infiltration 24-27. Although various immune cell populations are known to play roles in GBM, a detailed understanding of the immune response in GBM and the factors driving it remains incomplete. Therefore, we attempted to identify the correlation between immune cell infiltration and survival via the TIMER database. In GBM, the immune infiltration levels of regulatory T cells, neutrophil, macrophage, monocyte, dendritic cells, and cancer-associated fibroblasts are positively correlated with *IL-6* expression (Figure 2).

### Correlation of *IL-6* mRNA expression with patient survival

**Figure 3:**
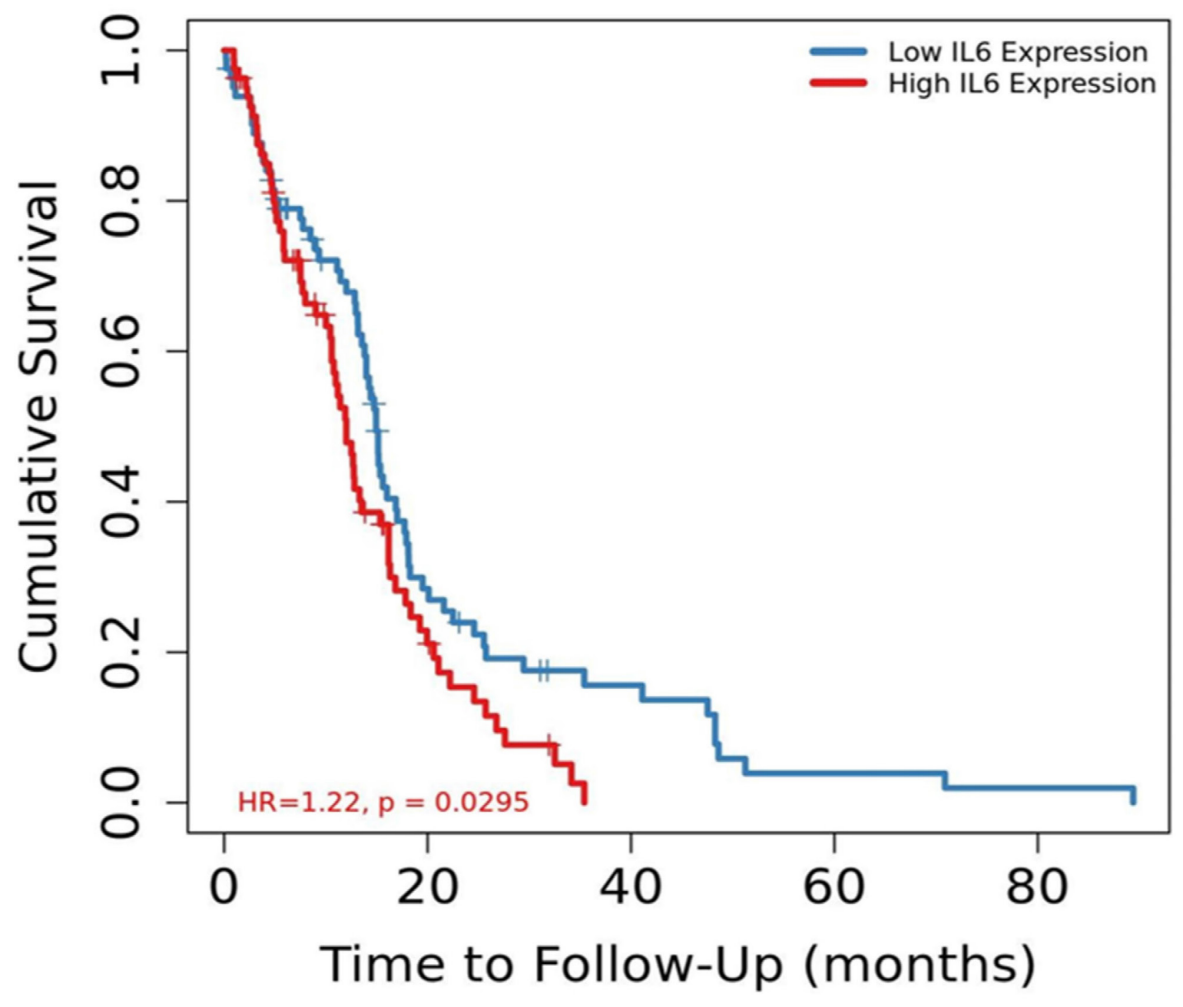
Kaplan-Meier survival curves comparing the high and low mRNA expression of *IL-6* in glioblastoma from TIMER database (p = 0.0295).

The GSEA results suggested that several pathways associated with cancer progression were upregulated in patients with GBM who had high *IL-6* mRNA expression. Therefore, the TIMER database, which stores gene expression data and clinical information was used to investigate whether the mRNA expression level of *IL-6* was a prognostic factor for GBM. The results revealed that patients with GBM who had high *IL-6* mRNA expression levels had a worse prognosis than those with low *IL-6* mRNA levels (Figure 3, p=0.0295, hazard ratio [HR] = 1.22). These findings suggest that *IL-6* expression levels could serve as indicators of prognosis in GBM patients.

### Patient characteristics and serum IL-6 test results in a clinical setting

**Figure 4:**
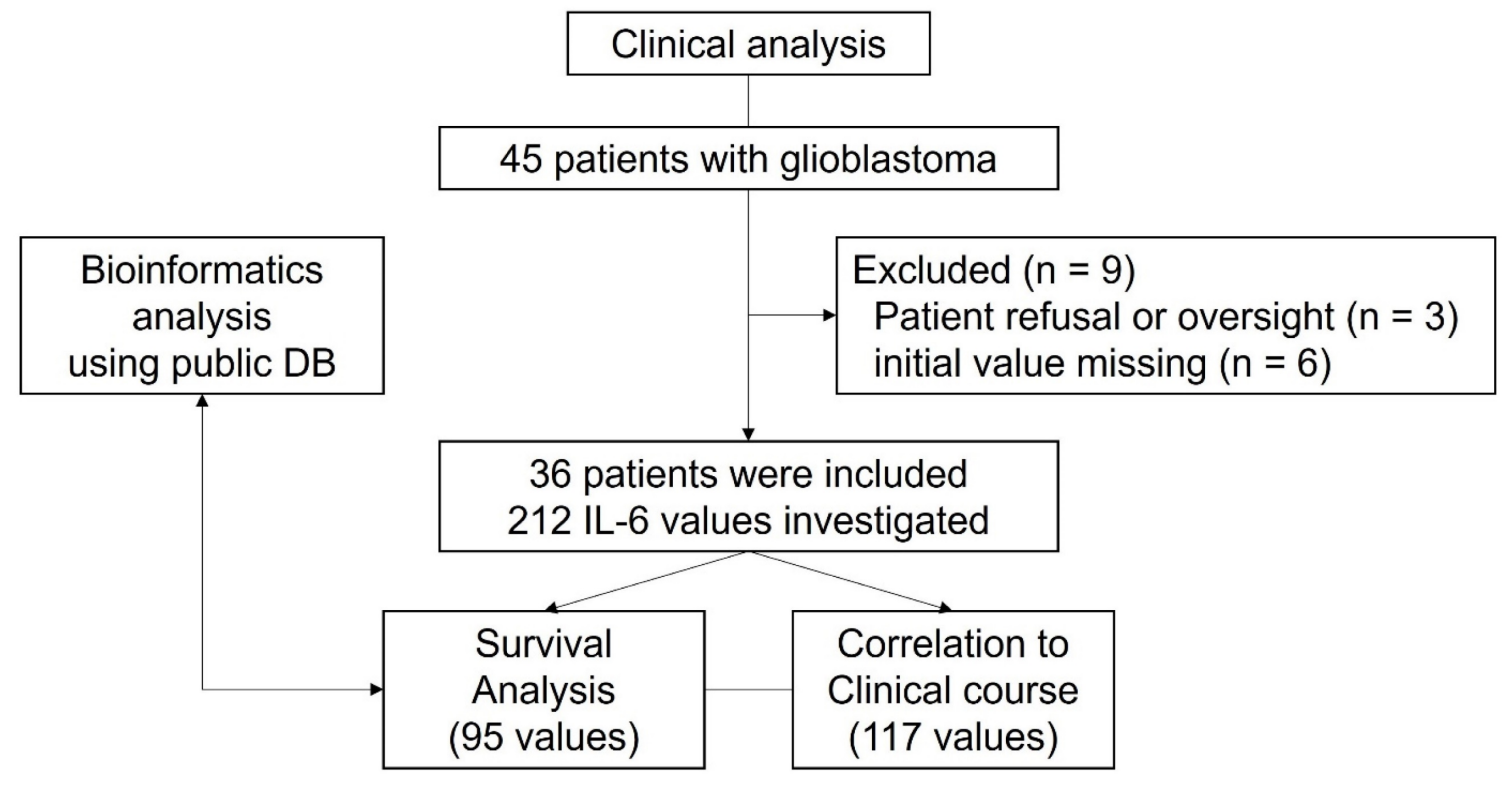
Study flow chart.

Between March 2021 and September 2023, 45 patients with glioblastoma who underwent surgery at a single tertiary institution were evaluated. Considering advantages of non-invasive biomarkers in predicting therapy response and prognosis, serum IL-6 levels were routinely measured in this cohort. However, three patients did not undergo serum IL-6 testing due to either refusal of the test or inadvertent omission. Six patients were also excluded because the initial value, which was not tested or superimposed with systemic inflammation, was missing. Consequently, 36 patients were analyzed in this study (Figure 4).

The median age was 67 years (range, 39-90 years), and 20 patients (55.6%) were male (Table 1). Favorable resection was achieved in 22 patients (61.1%), and unfavorable resection was performed in 14 patients (37.8%), including 4 biopsies only. MGMT methylation was confirmed in 14 patients (38.9%). Standard or short-course CCRT was performed in 31 patients (83.8%), and the median follow-up time was 12.5 months (range, 2-38.5 months). The results of the serum IL-6 concentration were obtained 305 times (range, 1.5-5000 pg/mL). Among these, 93 values were excluded because they were repeated within the same time interval or because they overlapped with other types of systemic inflammation. Therefore, 212 values at various time points were included in the analyses. The initial IL-6 value was obtained from all patients, with a median value of 5.6 pg/mL (range, 1.8-26.7 pg/mL). During the pre-CCRT period, 29 IL-6 values were obtained, with a median of 5.4 pg/mL (range, 1.5-14.5 pg/mL), whereas during the post-CCRT period, 30 values were confirmed, with a median of 3.4 pg/mL (range, 1.6-415 pg/mL). Elevated serum IL-6 levels (≥7 pg/mL) in the initial, pre-CCRT and post-CCRT time periods were confirmed in 11 of 36 (30.6%), 9 of 29 (31.0), and 6 of 30 (20.0%) patients, respectively. During adjuvant temozolomide treatment and thereafter, there was considerable variation between patients depending on treatment progress and survival status, but 117 values (range, 1.5-23.9 pg/mL) were available for analysis.

### Elevated serum IL-6 is related to survival time

**Figure 5:**
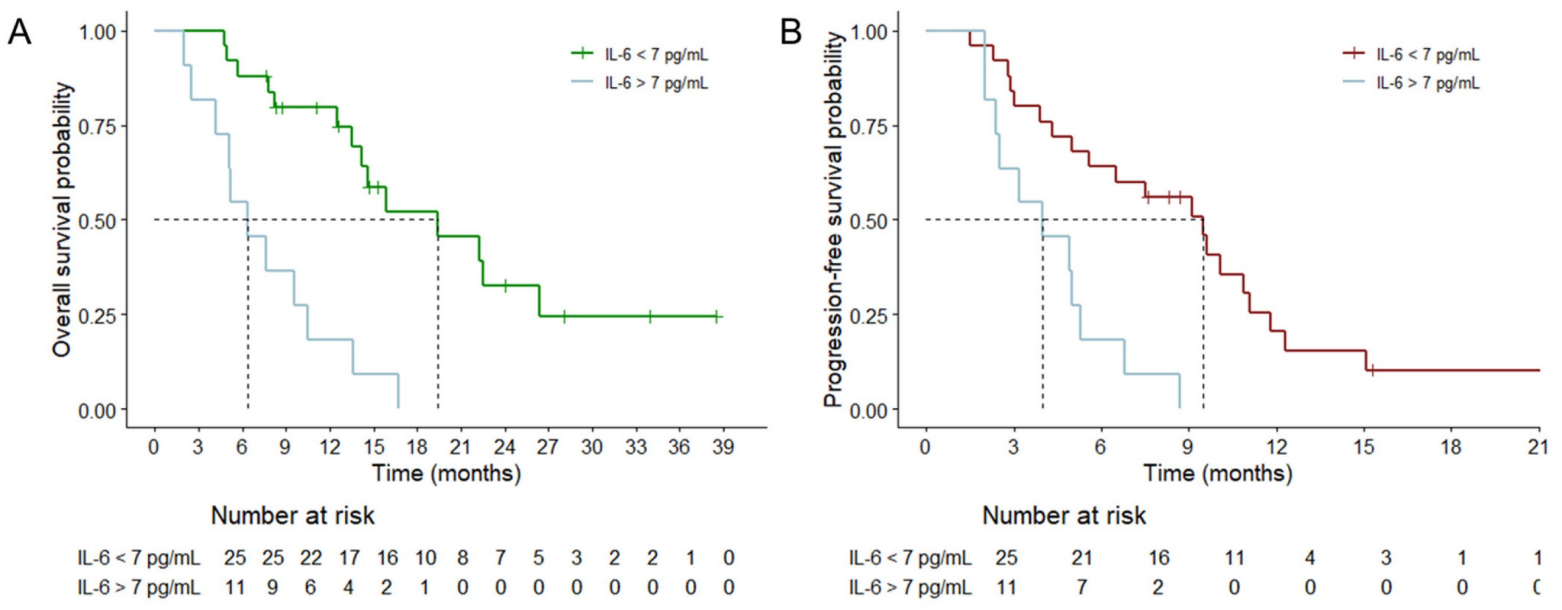
In a clinical setting, patients with high serum IL-6 levels (cutoff value of 7pg/mL) prior to surgery had significantly shorter overall survival (A) and progression-free survival (B) compared to those with low serum IL-6 levels.

At the time of analysis, 25 patients (69.4%) had reached the primary endpoint, and the median actuarial OS was 13.6 months (95% confidence interval [CI] = 11.1-16.1 months). In the multivariate analyses for shorter OS, elevated IL-6 was a significant risk factor (p = 0.048, HR = 2.749, and 95% CI = 1.711-9.626), as were unfavorable surgical resection (p = 0.039, HR = 2.755, 95% CI = 1.050-7.228) and an unmethylated MGMT status (p = 0.027, HR = 3.646, 95% CI = 1.161-11.448) (Table 2). Using an IL-6 level of 7.0 as the cutoff, patients were dichotomized into lower and higher initial IL-6 groups to compare survival curves (Figure 5A). The median actuarial OS of the lower initial IL-6 group (19.7 months, 95% CI = 10.3-28.4 months) was significantly longer than that of the higher initial IL-6 group (p < 0.001, 6.4 months, 95% CI = 3.67-16.1 months).

Tumor progression was evident in 31 patients (86.1%), and the median actual PFS was 5.6 months (95% CI = 3.0-8.2 months). In the univariate analyses for PFS, old age, unfavorable surgical resection, MGMT unmethylation, and initial IL-6 elevation were significant risk factors. However, in the multivariate analysis, surgical resection (p = 0.002) and the MGMT status (p < 0.001) were still significant risk factors, whereas old age and increased IL-6 were not significant risk factors. Nonetheless, when an IL-6 level of 7 was used as the threshold, the PFS curve revealed a significant difference between the lower and higher initial IL-6 groups (Figure 5B, p = 0.001, 9.5 vs. 4.0 months, respectively).

Although IL-6 values were not fully available for both the pre-CCRT and post-CCRT periods, it was confirmed that IL-6 elevation at these time points did not significantly affect OS or PFS (data not shown). Additionally, the trend of IL-6 and its correlation with clinical situations were analyzed via a multiple line graph, but this analysis was unsuccessful (supplementary Figure 1). Specifically, the clinical correlation was acceptable in only 13 patients (36.1%), while in 16 patients (44.4%), there was no relationship between IL-6 and tumor progression or pseudoprogression, and the relationship could not be evaluated in 7 patients.

## Discussion

In this study, we employed a data-driven approach to investigate the role of IL-6 in GBM via GSEA and the TIMER database. The results revealed that *IL-6* mRNA expression is associated with the activation of procancer pathways, immune cell infiltration and poor prognosis in GBM patients. To confirm the results of the bioinformatics analysis, we subsequently evaluated the prognostic significance of serial serum IL-6 levels in GBM patients in our hospital. The higher initial IL-6 group had poorer OS and PFS than the lower group did, highlighting its potential as a biomarker for guiding therapeutic strategies and predicting outcomes.

IL-6 is a key pleiotropic cytokine that influences immune and physiological reactions, including inflammation, the antigen-specific immune response, hematopoiesis, and cell differentiation 28. Dysregulation and elevation of IL-6 have been suggested to play significant roles in the development of inflammatory and autoimmune disorders as well as cancer 29. Several studies have revealed an inverse correlation between patient survival and serum IL-6 levels or mRNA expression in various types of tumors, including head and neck squamous cell carcinoma, esophageal cancer, gastric cancer, ovarian cancer and lung cancer 9, 11, 30-32. Additionally, low *IL-6* and *IL-6* receptor expression correlated with improved survival in the TCGA pancancer dataset^30^. Therefore, there is ongoing interest in the prognostic value of IL-6, and furthermore, targeted therapies for the IL-6 pathway are being developed^28^.

Since it has been confirmed that IL-6 and *IL-6* mRNAs are released by GBM both *in vitro* and *in vivo*, IL-6 is considered the key GBM-derived molecule that is often associated with poor prognosis 33, 34. Tachirkov *et al.* reported that *IL-6* gene amplification is associated with the aggressiveness of GBM and significantly correlated with decreased OS^35^, ^36^. The release of IL-6 by GBM promotes the recruitment of myeloid cells, which shifts the immune response from an inflammatory antitumor response to an anti-inflammatory, wound-healing-type response. This change reduces the effectiveness of immune cells in destroying tumor cells and can lead to tissue remodeling, creating an area of relative immune privilege that prevents immune access to tumor cells^37^. Additionally, IL-6 and other immunomodulatory molecules induce the activation of regulatory T cells, which suppress the antitumor T-cell response by releasing immunosuppressive cytokines such as IL-10 and transforming growth factor β. A recent study reported that GBM-associated endothelial cells are the major source of IL-6 in the GBM microenvironment and that IL-6 induces alternative macrophage activation and GBM progression 38. In this study, we confirmed through GSEA that IL-6 expression is associated with hypoxia and angiogenesis, which may be related to the proliferation of endothelial cells. Additionally, using the TIMER database, we observed that *IL-6* expression is also related to the infiltration of regulatory T cells and macrophages, which interact with IL-6 secretion in the TME. Futhermore, similar to our study, an analysis of data from the TCGA and Rembrandt databases revealed that elevated *IL-6* expression is correlated with poor OS in GBM patients 38.

Several authors have demonstrated the relationship between serum IL-6 levels and patient survival. Bunevicius *et al.* reported that an elevated IL-6 concentration, with a cutoff of 2.0 pg/mL, was significantly associated with shorter survival times in patients with high-grade glioma, with a HR of 4.068 39. Shan *et al.* reported a direct correlation between IL-6 levels in serum and cerebrospinal fluid and elevated IL-6 expression in glioma tissue. IL-6 values were significantly higher than in healthy controls and progressively increased with glioma grade 10. Furthermore, higher preoperative IL-6 (serum: > 20 pg/mL and CSF: > 2600 ng/mL) and a reduction in IL-6 after surgery were significantly correlated with worse OS 10. Gandhi *et al.* also supported the prognostic power of IL-6 in conjunction with other inflammation-related biomarkers, whereas Holst *et al.* reported that elevated serum IL-6 was associated with short OS in univariate analysis but not in multivariate analysis 40, 41.

Conversely, several studies have reported no effect of serum IL-6 levels or changes in survival, although these studies often had relatively small cohorts or included only recurrent GBM patients^42^-^44^. In the largest negative study conducted by Reyes, the serum IL-6 level of 47 GBM patients was not correlated with survival time. However, the study cohort had an unusually short OS of 8.1 months, with 32% of patients not receiving CCRT 45.

The postoperative clinical course is more variable than the preoperative state, with additional prognostic factors such as the EOR and surgical complications contributing to its complexity. In this study, establishing a correlation between IL-6 levels at various time points and tumor progression or prognosis was more challenging than analyses based solely on initial values. This difficulty likely stems from the intrinsic limitations of IL-6, which is highly susceptible to variations in systemic conditions. Similarly, Demirci *et al.* reported no correlation between serum IL-6 levels during the pre-CCRT period and PFS or OS 46. Therefore, there is a need for the development of standardized measurement protocols or auxiliary markers for IL-6 to increase its clinical utility in the future.

Several limitations are identified in our current study. First, the sample size is relatively small, which is not large enough to fully validate our results and determine clinical applicability of serum IL-6 to diverse cancer patients. Future studies may identify serum IL-6 level with stronger relationships to GBM prognosis. Second, this study was performed at a single center, which may lead to selection bias. The patient characteristics in this study may not be representative of GBM patients. Multicenter studies are required to validate our results. Third, although we found a correlation between initial IL-6 levels and patient outcomes, there is a lack of standardized protocols for measuring serum IL-6 levels, which can lead to variability in results. To date, although the role of IL-6 as a biomarker has been analyzed and reported through meta-analyses in many tumors, including colorectal and lung cancers, it lacks a clear cut-off value and shows high variability 11, 47. The cut-off value used in this study also requires further validation. Future studies should focus on developing standardized measurement protocols and investigating auxiliary markers to improve the reliability and clinical utility of serum IL-6. Therefore, larger, multicenter, prospective studies with standardized measurement protocols are essential to confirm our findings and to translate them into clinical practice. In addition, while we assumed a correlation between serum and tissue IL-6 expression based on previous studies, future experimental validation, especially focused on the role of TME, is necessary.

## Conclusions

In this study, bioinformatics analyses revealed a significant association between elevated *IL-6* mRNA expression and the activation of procancer pathways, increased immune cell infiltration, and poor prognosis in GBM patients. These findings were corroborated by clinical analyses of serum IL-6 levels in GBM patients, where higher initial IL-6 levels were linked to poor prognosis. Considering the prognostic values of IL-6 in GBM patients, measuring its serum level could potentially improve patient stratification, tailor therapeutic approaches, and enhance the overall management of GBM.

## Supplementary Material

Supplementary figure.

## Figures and Tables

**Table 1:** Patient characteristics

	Values
Median age (yrs, range)	67 (39-90)
Male (n, %)	20 (55.6)
Extent of Resection (n, %)	
Favorable	22 (61.1)
Supramaximal CE resection	3 (8.3)
Complete CE resection	13 (36.1)
Near-total CE resection	6 (16.7)
Unfavorable	14 (38.9)
Subtotal CE resection	7 (19.4)
Partial CE resection	3 (8.3)
Biopsy	4 (11.1)
MGMT methylation	
Methylated	14 (38.9)
Unmethylated	22 (61.1)
CCRT	
Not available	6 (16.7)
Standard	25 (69.4)
Short-course	5 (13.9)
Available serum IL-6 testing	
Total number*	212
Initial (preoperative)	36 (100)
Pre-CCRT (postoperative)	29 (78.4)
Post-CCRT	30 (81.1)
Adjuvant TMZ and thereafter	117

*overlapped with systemic inflammation and redundant values in the same periods were excluded CE = contrast enhancement, MGMT = O6-methylguanine-DNA methyltransferase, CCRT = concurrent chemoradiation therapy, IL-6 = Interleukin-6, TMZ = temozolomide

**Table 2:** Univariate and Multivariate risk factor analysis

Variables	Overall survival						Progression-free survival					
	Univariate			Multivariate			Univariate			Multivariate		
	HR	95% CI	p	HR	95% CI	p	HR	95% CI	p	HR	95% CI	p
Age >65	3.107	1.224-7.891	0.017	1.100	0.342-3.541	0.873	3.716	1.512-9.134	0.004	1.893	0.671-5.339	0.228
Male	0.800	0.358-1.785	0.585	1.567	0.626-3.922	0.338	0.645	0.315-1.323	0.232	0.753	0.322-1.764	0.514
Unfavorable resection	3.331	1.384-8.018	0.007	2.755	1.050-7.228	0.039	2.897	1.281-6.552	0.011	4.759	1.761-12.864	0.002
MGMT unmethylated	5.027	1.885-13.411	0.001	3.646	1.161-11.448	0.027	5.732	2.261-14.528	<0.001	7.013	2.492-19.741	0.000
Without CCRT	2.525	0.997-6.398	0.051	1.942	0.651-5.794	0.234	1.912	0.773-4.728	0.161	2.250	0.731-6.930	0.158
Initial IL6 > 7 pg /mL	5.316	2.200-12.846	<0.001	2.749	1.011-7.474	0.048	4.059	1.711-9.626	0.001	1.311	0.439-3.918	0.627

HR = hazard ratio, 95% CI = 95% confidential interval, MGMT = O6-methylguanine-DNA methyltransferase, CCRT = concurrent chemoradiation therapy, IL-6 = Interleukin-6
