# Supplementary material for: Elevated Serum IL-6 as a Negative Prognostic Biomarker in Glioblastoma: Integrating Bioinformatics and Clinical Validation
Source: J Cancer. 2025 Jan 1;16(3):802–11. doi: 10.7150/jca.104759 (PMC11705068; doi:10.7150/jca.104759)

Supplementary Figure 1. The trend of IL-6 with time and its correlation with clinical events, including tumor progression or pseudoprogression (red dots or black cross symbols); The presence of a clinical correlation was defined as follows: tumor progression was observed when there was an increase in IL-6, or pseudoprogression or stable disease was observed when there was no increase in IL-6. Green lines and red dots indicate correlated group and blue lines and black cross markers indicate unrelated group.

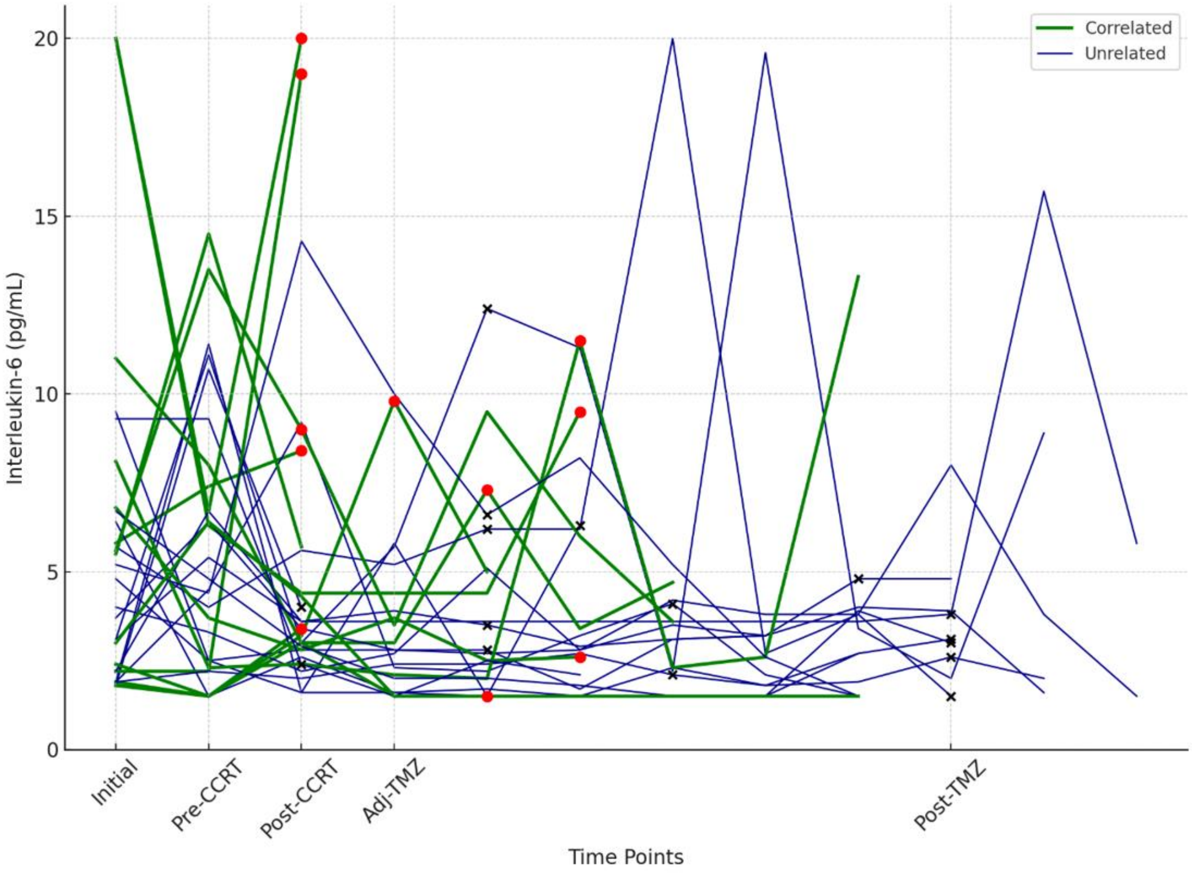

Supplement: Supplementary file 1 — Supplementary figure. [file jcav16p0802s1.pdf]
